# lncRNA SNHG15 Promotes Ovarian Cancer Progression through Regulated CDK6 via Sponging miR-370-3p

**DOI:** 10.1155/2021/9394563

**Published:** 2021-10-25

**Authors:** Yi Wang, Minghong Ding, Xiaoqing Yuan, Ruibao Jiao, Dagao Zhu, Wanzhong Huang, Wenxia Deng, Yulong Liu

**Affiliations:** ^1^Department of Oncology, The Second Affiliated Hospital of Soochow University, Suzhou 215004, China; ^2^Department of Oncology, Tongling People's Hospital, Tongling 244000, China; ^3^Department of Obstetrics and Gynecology, Tongling People's Hospital, Tongling 244000, China; ^4^Department of Pathology, Tongling People's Hospital, Tongling 244000, China; ^5^Department of Laboratory Medicine, Tongling People's Hospital, Tongling 244000, China; ^6^State Key Laboratory of Radiation Medicine and Protection, School of Radiation Medicine and Protection, Medical College of Soochow University, Suzhou 215123, China; ^7^Collaborative Innovation Center of Radiological Medicine of Jiangsu Higher Education Institutions, Suzhou 215123, China

## Abstract

Ovarian cancer is a kind of cancer from the female genital tract; the molecular mechanism still needs to be explored. lncRNA plays a vital role in tumorigenesis and development. Our aim was to identify oncogenic lncRNAs in ovarian cancer and explore the potential molecular mechanism. SNHG15 was initially identified by using GEO datasets (GSE135886 and GSE119054) and validated by tumor tissues and the cell line, identifying that SNHG15 was upregulated in ovarian cancer. Besides, high SNHG15 indicated poor prognosis in ovarian cancer. Furthermore, knockdown SNHG15 suppresses ovarian cancer proliferation and promotes apoptosis. Mechanistically, SNHG15 promotes proliferation through upregulated CDK6 via sponging miR-370-3p. Taken together, our findings emphasize the important role of SNHG15 in ovarian cancer, suggesting that SNHG15 may be a promising target for ovarian cancer.

## 1. Introduction

Ovarian cancer is the leading fatal neoplasm of the female genital tract [1–3]. Ovarian cancer affects annually 295414 new patients, with approximately 184799 deaths/year [4]. Despite the rapid development of diagnosis and treatment, the 5-year survival remains poor [5, 6]. Therefore, the mechanism of ovarian cancer occurrence and new strategies of treatment still need to be explored.

Long noncoding RNAs (lncRNAs) are non-protein-coding RNAs with a length of more than 200 nucleotides [7]. With the deepening of research in recent years, people's cognition of lncRNAs has been consistently improved and a large amount of evidence suggests that lncRNA has become an indispensable participant in the development of different human tumors [8–10]; for instance, HOTAIR promotes hepatocellular carcinoma progression [11] and regulated leukemia differentiation [12]; H19 promotes glioma angiogenesis [13] and promotes leukemogenesis [14]; MEG3 inhibits prostate cancer progression [15] and regulates imatinib resistance in chronic myeloid leukemia [16]. Therefore, lncRNA may be a promising therapeutic target in treating tumors, including ovarian cancer; there is a great need to identify lncRNAs to provide a new treatment strategy for ovarian cancer.

Recently, SNHG15 was described as an oncogenic lncRNA in several cancers [17–19] but the role of SNGH15 in ovarian cancer remains unclear. Herein, SNHG15 was identified by using GEO datasets and validated by ovarian cancer tissues and we investigated the clinical value and mechanism of SNHG15 in ovarian cancer. We hope that this research may provide novel ideals for clinical-targeted therapy of ovarian cancer.

## 2. Methods

### 2.1. Databases and Bioinformatics Analysis

We identified 2 datasets (GSE135886 and GSE119054) by using the keywords: “ovarian cancer and lncRNA” in the Gene Expression Omnibus (GEO) database (https://www.ncbi.nlm.nih.gov/gds/). The GEO2R tool was used to screen for differentially expressed lncRNAs (DElncRNAs) between healthy ovarian tissue and ovarian cancer tissue. DElncRNAs were selected as the following criteria:|log2FC | >2 and *P*_adj_ value < 0.05.

Gene Expression Profiling Interactive Analysis (GEPIA) database (http://gepia.cancer-pku.cn) was employed to analyze the clinical significance of SNHG15.

Kaplan-Meier plotter database (http://kmplot.com/) was employed to explore the prognostic value of SNHG15 in ovarian cancer patients.

StarBase database (http://estarbase.sysu.edu.cn) was employed to identify the relationship between SNHG15, miR-370-3p, and CDK6.

### 2.2. Patients' Samples

Ovarian cancer and normal tissues from newly diagnosed ovarian cancer patients in our hospital from 2019 to 2021 were collected. The study was conducted according to the principles of the Declaration of Helsinki, approved by the medical ethics committee of Tongling People's Hospital, and with the written informed consent of each patient.

### 2.3. Cell Line Culture and Transfection

SKOV3 and IOSE80 were purchased from the Shanghai cell bank of Chinese Academy of Sciences. Cells were cultured in DMEM medium (Gibco) supplemented with 10% fetal bovine serum and cultured at 37°C in a 5% CO_2_ incubator.

miRNA negative control (NC), the miR-370-3p inhibitor, si-SNHG15, and si-NC were ordered from GenePharma (Shanghai, China). Lipofectamine 2000 (Thermo Fisher Scientific Inc.) was used to transfected these into cell according to the manufacturer's protocol.

### 2.4. Quantitative Real-Time PCR

RNA from cells were extract by using TRIzol reagent (Invitrogen), and the PrimeScript RT reagent kit (TaKaRa, Dalian, China) was used to synthesize cDNA according to the manufacturer's protocol. qRT-PCR was performed by using TB Green PCR Mix (TaKaRa, Dalian, China). The primer sequences used were SNHG15-F: GGTGACGGTCTCAAAGTGGA, SNHG15-R: GCCTCCCAGTTTCATGGACA, GAPDH-F: GGAGCGAGATCCCTCCAAAAT, and GADPH-R: GGCTGTTGTCATACTTCTCATGG; the miRNA reverse transcription PCR and qRT-PCR primers were ordered from RiboBio Inc. (Guangzhou, Guangdong, China). The expression of lncRNA was analyzed by 2^−ΔΔ*Ct*^ method.

### 2.5. Flow Cytometry

For apoptosis, each group of cells was collected and washed twice with PBS, using an apoptosis detection kit (BD Biosciences, Bedford, MA, United States), according to the manufacturer's protocol.

For cell cycle, each group of cells was collected and fixed with 70% precooled ethanol, using a cell cycle detection kit (Beyotime Institute of Biotechnology, China), according to the manufacturer's protocol.

Cell cycle and cell apoptosis were detected by CytoFLEX (Becton Dickinson, USA) and cell cycle was analyzed by Kaluza software (Kaluza® Analysis Software, Beckman Coulter).

### 2.6. Cell Proliferation and Scratch Assay

Each group of cells was measured at 24, 48, and 72 hours after cell transfection by CCK8 assay (Beyotime Institute of Biotechnology, China), according to the manufacturer's protocol.

Each group of cells was seeded in a 6-well plate, and when the bottom confluence reached 80%, scratch the bottom of the well with 100 *μ*L sterile pipette tip. The healed wounds were imaged at 0 and 48 h after scratching.

### 2.7. Luciferase

Cells were seeded and cotransfected with SNHG15-WT/SNHG15-MT or CDK6-WT/CDK6-MT vector and NC or the miR-370-3p inhibitor using Lipofectamine 2000 in 24-well plates. Vectors are based on psicheck2 and the binding sites of miR-370-3p were constructed. 48 hours later, luminescence was detect by using the Luciferase Reporter Assay Kit (Beyotime Institute of Biotechnology, China) according to the manufacturer's protocol.

### 2.8. Western Blot

Cells were lysed with RIPA containing 1% PMSF (Beyotime Institute of Biotechnology, China) and then clarified by centrifugation. The proteins were electrophoresed by SDS-PAGE and then transferred into PVDF membranes. CDK6 (1 : 5000) and *β*-actin (1 : 3000) (Cell Signaling, Danvers, MA, USA) were the primary antibodies. Chemiluminescent signal was detected by ECL staining (Beyotime Institute of Biotechnology, China).

### 2.9. Statistical Analysis

All the experiment results were analyzed by GraphPad Prism 6.0 software (GraphPad Software, San Diego, CA). *P* value < 0.05 was considered statistically significant.

## 3. Results

### 3.1. SNHG15 Was Upregulated and Indicated Adverse Prognosis of Ovarian Cancer

**Figure 1:**
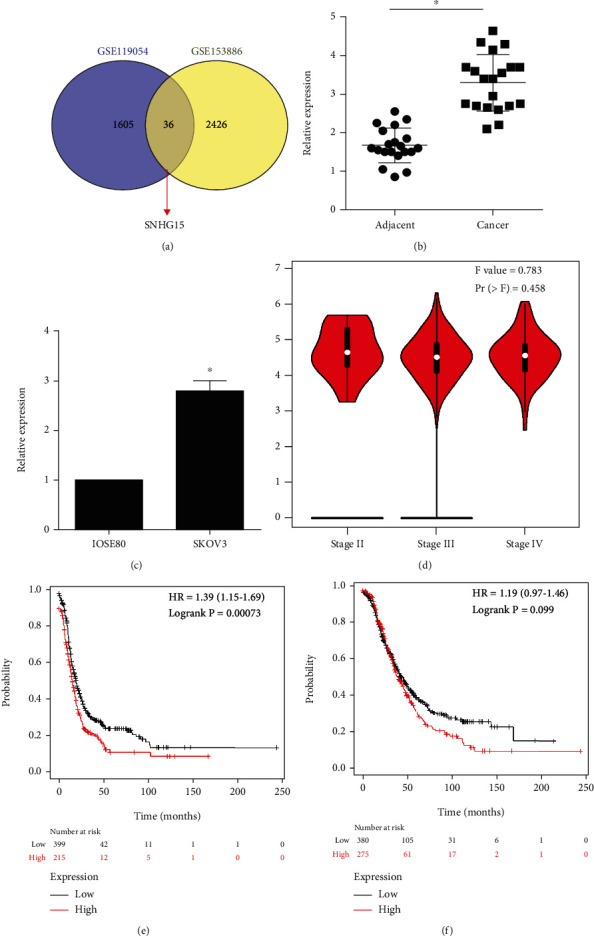
The identification of SNHG15 and the clinical value of SNHG15. Identification of SNHG15 by GEO datasets (a); SNHG15 upregulated in ovarian cancer tissues (b) and cancer cell line (c); GEPIA database indicated that SNHG15 did not correlate with the clinical stage in ovarian cancer (d); Kaplan-Meier plotter database indicated that SNHG15 correlated with PFS (e) but not OS (f).

To identify abnormally expressed lncRNA in ovarian cancer, we employed GSE135886 and GSE119054 from the GEO database to explore potential oncogenic lncRNA. As shown in Figure 1(a), there were 1605 lncRNAs upregulated in the GSE119054 dataset and 2426 lncRNAs upregulated in the GSE153886 dataset. There were 36 lncRNA in both datasets, and SNHG15 was among them.

To validated the aberrant expression of SNHG15, we performed qPCR to assess the SNHG15 expression in 20 patients, and highly expressed SNHG15 were observed in cancer tissues (Figure 1(b)) and SKOV3 cells (Figure 1(c)).

To evaluated the clinical significance of SNHG15, GEPIA was used to analyze the correlation between SNHG15 and the clinical stage of ovarian cancer; however, SNHG15 did not correlate with the clinical stage (Figure 1(d)). Simultaneously, we used Kaplan-Meier plotter database to explore the relationship between SNHG15 and survival. As shown in Figure 1(e), high SNHG15 predicted poor PFS. For OS, there was a strong trend that was not statistically significant (Figure 1(f)). Above all, high SNHG15 predicted poor prognosis in ovarian cancer.

### 3.2. SNHG15 Knockdown Suppressed the Proliferation of Ovarian Cancer Cell

**Figure 2:**
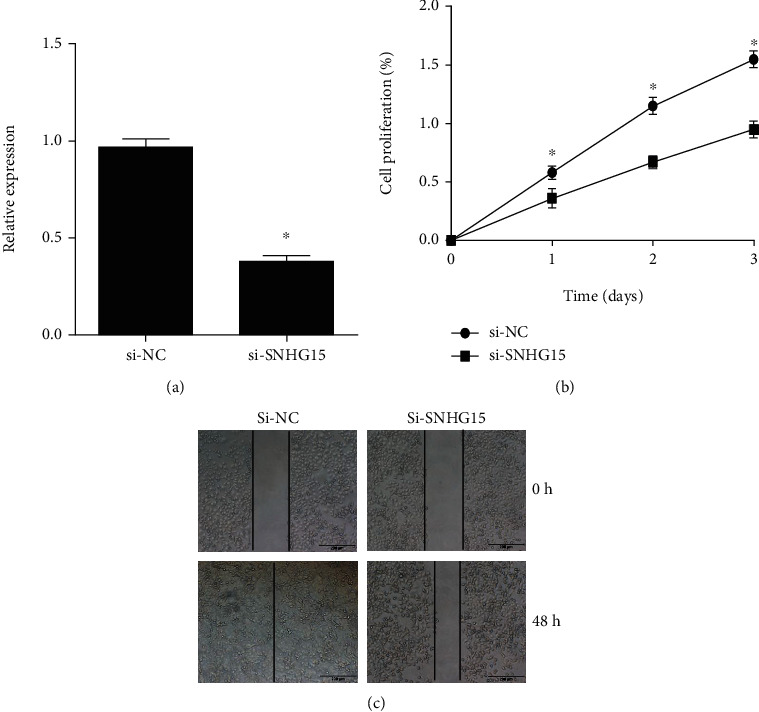
The impact of SNHG15 on the proliferation and migration of ovarian cancer. SNHG15 was significantly downregulated after siRNA transfection (a); SNHG15 suppressed the proliferation (b) and migration (c) of ovarian cancer cell. ^∗^*P* < 0.05.

In summary, SNHG15 was upregulated and predicted poor prognosis in ovarian cancer but the mechanism of SNHG15 remains undetermined. We transfected SKOV3 cells with the specific siRNA target SNHG15 (Figure 2(a)). CCK8 assays were employed to assess the impact of SNHG15 on the proliferation of SKOV3 cell; as shown in Figure 2(b), the cell proliferation rate decreased in the si-SNHG15 group than that in si-NC group.

In addition, the scratch test was used to assess the impact of SNHG15 on the migration of SKOV3 cell, as shown in Figure 2(c); in the si-SNHG15 group, the scratch area healing rate after 48 hours was lower than that in the si-NC group. These results indicated that SNHG15 knockdown restrained the migration and proliferation of ovarian cancer cell.

### 3.3. SNHG15 Knockdown Arrest Cell Cycle and Promoting Apoptosis of Ovarian Cancer Cell

**Figure 3:**
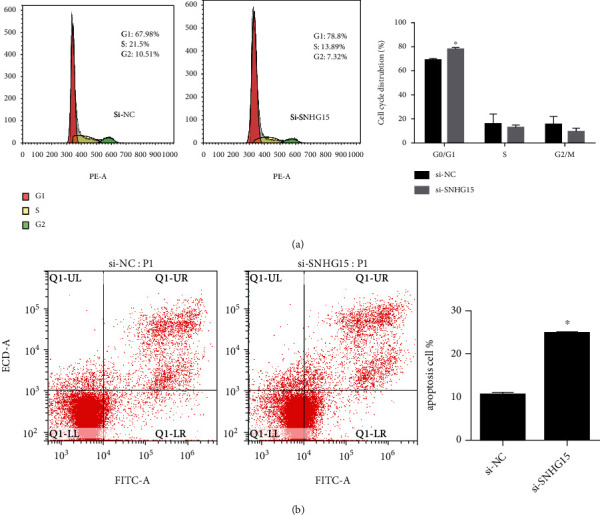
Effect of SNHG15 on cell cycle and apoptosis of ovarian cancer. SNHG15 knockdown induces G0/G1 arrest (a) and promotes apoptosis (b). ^∗^*P* < 0.05.

Then, we investigated the impact of SNHG15 on cell cycle and apoptosis. And our results indicated that the proportion of the G0/G1 phase was higher in the si-SNHG15 group than the si-NC group (Figure 3(a)), indicating that SNHG15 knockdown induced G1/G0 phase arrest.

Besides, flow cytometry revealed an increase in apoptosis in the si-SNHG15 group than the si-NC group (Figure 3(b)), indicating that SNHG15 knockdown promoted ovarian cancer cell apoptosis.

### 3.4. SNHG15 Regulated CDK6 Expression

**Figure 4:**
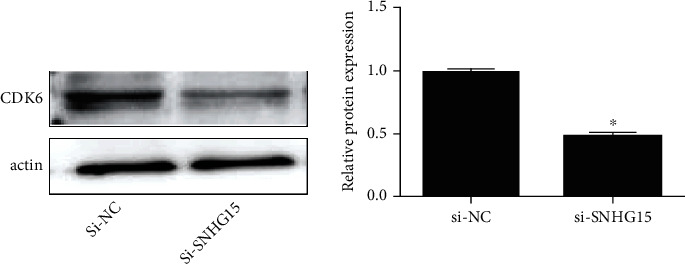
The impact of SNHG15 on CDK6 expression of ovarian cancer.

CDK6 regulated cell cycle progression under physiological and pathological conditions [20, 21], and published evidences have showed that CDK6 was important in the development of ovarian cancer [22, 23]. To demonstrate whether SHNG15 regulated CDK6 in ovarian cancer, we analyzed the CDK6 expression after SNHG15 knockdown, and in the si-SNHG15 group, CDK6 was lower than that in the si-NC group (Figure 4), indicating that SNHG15 regulated CDK6 expression in ovarian cancer.

### 3.5. SNHG15 Regulated CDK6 Expression via miR-370-3p

**Figure 5:**
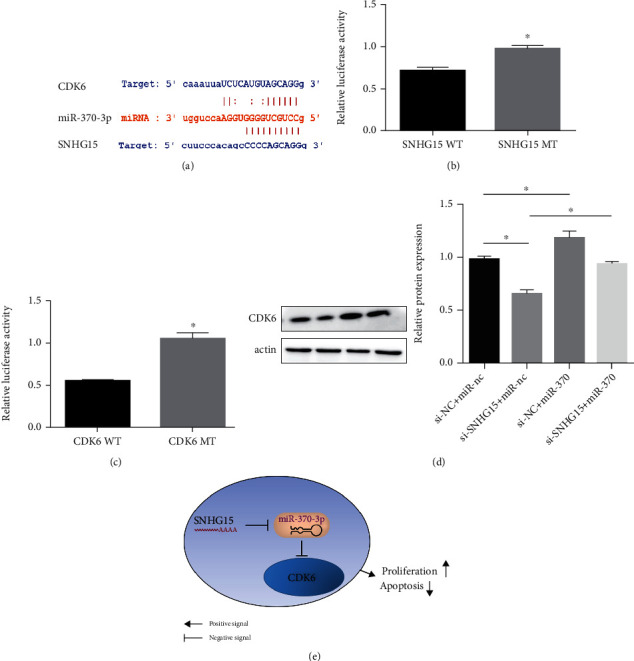
SNHG15 regulated CDK6 via sponging miR-370-3p. The predicted binding site of SNHG15, miR-370-3p, and CDK6 (a). The predicted binding sites of miR-370-3p to SNHG15 (b) and CDK6 (c) were confirmed by luciferase reporter assay. The miR-370-3p inhibitor restored downregulated expression of CDK6 induced by SNHG15 knockdown (d); the hypothetical mechanism of SNHG15 in ovarian cancer (e).

SNHG15 usually regulated the expression of target gene via miRNA in cancers [24, 25]. Thus, we used StarBase database to predict potential miRNA that interacts with SNHG15 and CDK6. miR-370-3p was suggested as a potential miRNA which interacts with SNHG15 and CDK6 (Figure 5(a)). Subsequently, we construct plasmids containing the wild-type and mutated-type binding sites of miR-370-3p in SNHG15 and 3′UTR of CDK6 for luciferase report assays. And the results of luciferase report assays showed that luciferase activity was decreased in the SNHG15 wild-type and CDK-3′UTR wild-type groups than the SNHG15 mutated-type and CDK-3′UTR mutated-type groups (Figures 5(b) and 5(c)). Lastly, rescue experiment indicated that the miR-370-3p inhibitor restored downregulated expression of CDK6 induced by SNHG15 knockdown (Figure 5(d)). To sum up, our results demonstrate that SNHG15 plays a biological role in ovarian cancer through CDK6 via miR-370-3p (Figure 5(e)).

## 4. Discussion

A growing body of evidences suggests the association of tumor and lncRNA, and lncRNA may be a therapeutic target for tumor [26, 27]. Herein, we initially identified that SNHG15 was upregulated in ovarian cancer based on published data, and then, we validated it in tumor tissues and cell lines. Lastly, the clinical value and mechanism of SNHG15 in ovarian cancer were investigated. The results showed that SNHG15 was upregulated and predicted poor prognosis in ovarian cancer; in addition, SNHG15 promoted cancer cell proliferation through upregulated CDK6 via inhibited mir-370-3p.

SNHG15 was firstly reported in 2012 [28]. Subsequently, SNHG15 was investigated in tumor, and the SNHG15 expression was upregulated in cancers and high SNHG15 expression predicted poor prognosis, for instance, gastric cancer [17], lung cancer [18], colorectal cancer [29], and glioma [24]. Consistent with the recent evidence raised by Zhang et al. [30], our results showed that SNHG15 was upregulated in ovarian cancer; it is noteworthy that our results were based on the GEO dataset and verified by the specimen of our own; this makes the results more reliable. In addition, the prognostic role of SNHG15 was identified by Kaplan-Meier plotter database due to our limited sample size and SNHG15 was correlated with poor PFS. Nonetheless, these results indicated that SNHG15 was highly expressed in ovarian cancer and predicted poor outcome; it can be used to stratify newly diagnosed patients and initiate individualized therapeutic methods.

According to published data, SNHG15 promotes tumor progression via serving as miRNA sponges to inhibit miRNA function, implying that in papillary thyroid carcinoma, SNHG15 controlled the YAP1-Hippo signaling pathway via miR-200-3p [25], and accelerates tumor progression in liver cancer via inhibiting miR-141-3p [31] and via sponging miR-338-3p in colorectal cancer [19]. In our study, we found that SNHG15 silencing suppresses proliferation of ovarian cancer, whilst inducing G0/G1 phase arrest and promoting apoptosis. What is more, the G1/S checkpoint regulator—CDK6—expression was downregulated, which suggests that CDK6 was the target of SNHG15. Indeed, a recent study suggests that SNHG15 targets CDK6 in glioma [24]. Then, we identified miR-370-3p as the bridge between SNHG15 and CDK6 by using online database, luciferase report assays, and rescue experiment. Taken together, our results showed that SNHG15 promoted ovarian cancer cell proliferation through upregulated CDK6 via inhibiting mir-370-3p.

## 5. Conclusion

In conclusion, our study suggests that SNHG15 may be used as a prognostic indicator in ovarian cancer and reveals the SNHG15/miR-370-3p/CDK6 pathway in ovarian cancer. Our findings provided a new molecular mechanism of SNHG15 in ovarian cancer and may provide theoretical basis for the development of new clinical treatment strategies.

## Data Availability

The datasets used and/or analyzed during the current study are available from the corresponding author upon reasonable request.

## Abbreviations

CCK8: Cell counting kit-8
DElncRNAs: Differentially expressed lncRNAs
GEO: Gene Expression Omnibus
FC: Fold change
GEPIA: Gene expression profiling interactive analysis
lncRNAs: Long noncoding RNAs
OS: Overall survival
PFS: Progression-free survival.
